# Spectroscopic Analysis of the Extracellular Matrix Hierarchical Structure in Naked Mole-Rat Skin

**DOI:** 10.3390/gels12040303

**Published:** 2026-04-01

**Authors:** Tetsuya Adachi, Hayata Imamura, Risa Tamagawa-Mineoka, Toyonari Yaji, Makoto Kawano, Shigenori Itsuzaki, Keiji Adachi, Fumishige Oseko, Shunichi Shibata, Satoru Shindo, Sachiro Kakinoki, Osam Mazda, Toshihisa Kawai, Kyoko Miura, Wenliang Zhu, Giuseppe Pezzotti

**Affiliations:** 1 Department of Dental Medicine, Graduate School of Medical Science, Kyoto Prefectural University of Medicine, Kamigyo-ku, Kyoto 602-8566, Japan; 2 Department of Immunology, Graduate School of Medical Science, Kyoto Prefectural University of Medicine, Kamigyo-ku, 465 Kajii-cho, Kyoto 602-8566, Japan; 3 Ceramic Physics Laboratory, Kyoto Institute of Technology, Sakyo-ku, Matsugasaki, Kyoto 606-8585, Japan; 4 Department of General Allergy Center, Fujita Health University, Dengakugakubo, Kutsukake-cho, Toyoake 470-1192, Japan; 5 Synchrotron Radiation Center, Ritsumeikan University, 1-1-1 Noji-Higashi, Kusatsu 525-8577, Japan; 6 Kawano Laboratory, Inc., Room 201, Incubation Building, The Institute of Scientific and Industrial Research (ISIR), Osaka University, 8-1 Mihogaoka, Ibaraki 567-0047, Japan; 7 Sensor Equipment & Medical Device Division, TATSUTA Electric Wire & Cable Co., Ltd., 3-17 Osadano-cho, Fukuchiyama City 620-0853, Japan; 8 Department of Anatomy, School of Dentistry, Health Sciences University of Hokkaido, 1757 Kanazawa, Tobetsucho, Ishikari 061-0293, Japan; sshibataanat@hoku-iryo-u.ac.jp; 9 College of Dental Medicine, Nova Southeastern University, 3200 South University Drive, Fort Lauderdale, FL 33328, USA; sshindo1@nova.edu (S.S.);; 10 Department of Chemistry and Materials Engineering, Kansai University, 3-3-35 Yamate-cho, Suita-shi 564-8680, Japan; 11 Department of Stem Cell Biology and Medicine, Graduate School of Medical Sciences, Kyushu University, 3-1-1 Maidashi, Fukuoka 812-8582, Japan; 12 Department of Aging and Longevity Research, Faculty of Life Sciences, Kumamoto University, Kumamoto 860-8556, Japan; 13 Biomedical Engineering Center, Kansai Medical University, 1-9-11 Shin-machi, Hirakata 573-1191, Japan

**Keywords:** hyaluronic acid, naked mole-rats, Raman spectroscopy

## Abstract

Naked mole-rats are extremely long-lived rodents with a lifespan of up to 40 years, during which cellular and tissue aging is rarely observed. In this study, we analyzed the extracellular matrix (ECM) of naked mole-rat skin at the molecular level to elucidate the molecules involved in anti-aging and their localization. Raman spectroscopy and Fourier transform infrared spectroscopy were applied to investigate the hierarchical structure of the ECM, showing that, whereas the epidermis of aged mice had thinned, the epidermis of naked mole-rats became thickened and hyaluronic acid (HA) was distributed under the basement membrane. Furthermore, naked mole-rat skin had a regular skin texture and flexibility, allowing the maintenance of a youthful appearance. Hyaluronic acid in naked mole-rats characteristically exists as clusters (chain HA) in skin tissue, where it is thought to permit moisture retention and maintain elasticity, contributing to the skin’s youthful appearance. These results suggested that not only the density of ECM but also its spatial distribution and topographic properties are important for skin anti-aging. Our findings may contribute to the elucidation of skin disease pathology, the development of therapeutic gel scaffolds, and the control of aging.

## 1. Introduction

The face is the core of our identity and the most obvious differences seen with age are the development of facial wrinkles and skin sagging. As we age, the hierarchical structure of the skin changes, causing the dermis to lose elasticity and contractile power, which leads to its hardening, wrinkling and sagging.

The skin is the largest organ in the human body and has a structure consisting, from the outside in, of the epidermis, dermis, and subcutaneous tissue layers. The epidermis is an epithelial tissue with a multilayered structure; its top layer is a dead cell layer called the stratum corneum. This layer has been considered unnecessary, as “dirt,” and has not been the object of much study.

The stratum corneum is formed through a special type of cell death (corneoptosis) of granulosa cells (SG1) in the uppermost part of the granular layer [1]. It was acquired when amphibians moved onto land in the late Devonian period and is, therefore, a structure present in terrestrial vertebrates such as amphibians, reptiles, birds, and mammals. This layer is susceptible to damage by external stimuli (such as ultraviolet rays) and foreign substances (allergens, etc.), but functions as a barrier to prevent invasion by bacteria and viruses. This barrier function prevents allergens (mites, food, etc.), bacteria, and viruses from entering the body, and stops water loss through the skin. The middle layer of the stratum corneum is acidic and is said to function as a defense against bacterial invasion [2].

The skin is an organ that changes with age. Facial skin, in particular, is prone to age-related wrinkling and sagging, so skin that is lustrous and firm gives the impression that people are younger than they actually are.

Hyaluronic acid (HA) is found in large amounts in the dermis, between cells, where it protects cells by retaining moisture and acting as a cushion.

Furthermore, intercellular lipids composed of ceramides, fatty acids, and cholesterol play a cement-like role to firmly adhere stratum corneum cells together, and act as a barrier by preventing moisture within the skin from evaporating.

However, with age, the thickness of the stratum corneum layer and the level of natural moisturizing factors decrease, causing skin dryness, which further progresses dryness, leading to cracking and inflammation. Therefore, controlling aging and maintaining the barrier structure aids in the prevention of skin diseases.

Skin aging research requires looking comprehensively at the extracellular matrix (ECM) that constitutes not only the dermis but also the epidermis.

In this study, we focused on naked mole-rats, an extremely long-lived rodent with a maximum lifespan of 40 years. Naked mole-rats have been reported to exhibit remarkably slow aging and reduced age-associated functional decline in tissues or organs [3]. Elucidating the unique physiological functions of naked mole-rats, including their remarkable resistance to aging, is extremely important for developed countries that are facing a super-aging society, which needs to control some consequences of aging. It is known that high-molecular-weight HA (6–10-fold larger than human HA), which is an ECM, has been proposed to contribute to their resistance against aging and cancer [4,5].

The in vivo dynamics of ECM constituent molecules remain unknown. Visualization of the ECM and the establishment of a method for doing so would contribute to new anti-aging developments.

Raman spectroscopy and Fourier transform infrared (FTIR) spectroscopy allow non-destructive, non-invasive analysis of molecular structures, whereas optical coherence tomography (OCT) enables non-destructive, non-invasive observation of the topographic structure of skin texture in three dimensions.

Therefore, in this study, we combined spectroscopic analysis, which visualizes molecular structure, with OCT, which analyzes topographic properties, to simultaneously analyze the quality, spatial information, and physical properties of various ECM constituent molecules in the skin (glycans, lipids, hydrated and water, etc.), to clarify the molecular mechanisms of anti-aging and the development of therapeutic gel scaffolds.

## 2. Results and Discussion

### 2.1. Histological Findings

**Figure 1:**
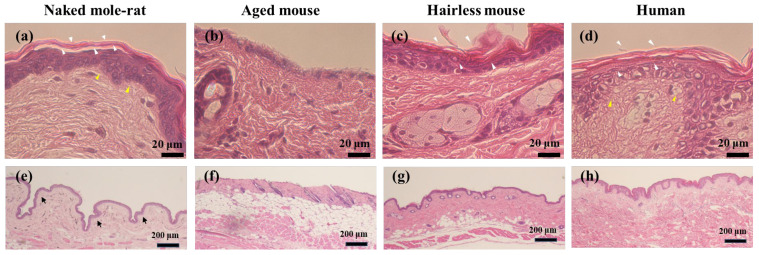
Representative micrographs of the H&E-stained skin tissue. White arrowheads indicate stratum corneum. Yellow arrowheads and black arrows indicate basement membrane. (**a**–**d**) High magnification. (**e**–**h**) Low magnification.

Histology of skin samples showed that epidermal thickness was maintained in the naked mole-rat (Figure 1a). By contrast, thinning of the epidermis observed in aged mice was considered to be a typical change associated with aging (Figure 1b), similar to that seen in humans.

It was confirmed that the stratum corneum of the naked mole-rats was maintained, whereas that of aged mice was partially lost because the stratum corneum functions as an epithelial barrier, and its loss is a typical symptom of aging. Neither hairless mouse skin (7-week-old; Figure 1c) nor human skin (28-year-old; Figure 1d) showed thinning of the epidermis, nor were any signs of aging, such as detachment of the stratum corneum, observed.

**Figure 2:**
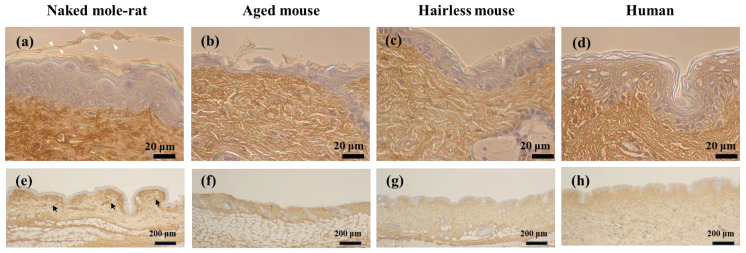
Light microscope images of the skin tissue stained with biotinylated hyaluronic acid (HA)-binding protein. White arrowheads indicate stratum corneum. Black arrows indicate HA just below the basement membrane. (**a**–**d**) High magnification. (**e**–**h**) Low magnification.

While the basement membrane of aged mice was flat (Figure 1f), that of naked mole-rats was uneven (Figure 1e). It is known that, when there is active turnover and a large number of epidermal stem cells, the basement membrane becomes wavy, suggesting that naked mole-rat skin has active turnover [6,7]. From immunohistochemistry findings it was confirmed that, in the naked mole-rat, a large amount of HA was present under the basement membrane of the dermis (strong reaction) compared with other species (Figure 2a,e). The stratum corneum of naked mole-rats expressed HA, consistent with findings reported by Kulaberoglu et al. [8]. In contrast, HA was ubiquitously expressed in the dermis of aged mice (Figure 2b,f).

The most interesting findings for the naked mole-rat epidermis suggest delayed skin aging in the naked mole-rats. Keratinocytes that made up the epidermis were large in the naked mole-rat, had abundant cytoplasm and contained large bright nuclei. This imaging is typical of cells undergoing vigorous activity (protein synthesis, etc.). The naked mole-rat skin appeared youthful compared with that of aged mice.

**Figure 3:**
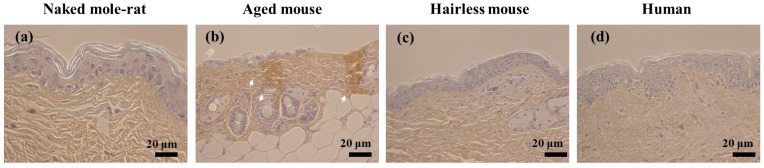
Light microscope images of the skin tissue stained with biotinylated collagen hybridizing peptide (CHP). White arrows indicate collagen degeneration. (**a**) Naked mole-rat; (**b**) Aged mouse; (**c**) Hairless mouse; (**d**) Human.

Collagen hybridizing peptide (CHP) is a novel and unique peptide that specifically binds unfolded collagen chains [9,10]. In naked mole-rats, staining with a CHP probe did not detect denatured collagen (Figure 3a), but did detect collagen degeneration in the dermis of aged mice (Figure 3b). Decreased production and degeneration of collagen by fibroblasts in the dermis have been reported to accelerate skin aging [11].

### 2.2. FTIR

To analyze the details of the molecular structure of the skin tissues, two types of spectroscopic analyses, FTIR spectroscopy and Raman spectroscopy, were performed on the investigated skin samples.

FTIR spectroscopy can analyze the water content of the stratum corneum, natural moisturizing factor (NMF), free fatty acids (FFAs), and structural proteins (β-sheets and α-helices) [12].

Raman spectroscopy permits the non-invasive evaluation of the distribution and changing behavior of skin components in a “living” state. Egawa et al. reported the visualization of the response of skin components, such as water, proteins, and lipids, to the external environment, as well as the detailed process of epidermal differentiation related to skin metabolism [13].

Miyamori et al. identified age by spectroscopic analysis of the secondary structure of proteins in skin tissue in a state close to the living body without conventional pretreatment [14]. To date, this analysis technology has made it possible to understand the qualitative changes (senescence state) of collagen due to aging by analyzing the secondary structure of proteins.

However, because FTIR spectroscopy and Raman spectroscopy differ in the molecule–light interactions they can detect and their resolution, it is believed that more detailed analysis can be performed by analyzing the changes in both molecular vibrations, such as the functional groups and skeletal structures of molecules to which IR spectroscopy and Raman spectroscopy are sensitive, respectively [15].

As described above, Raman spectroscopy and FTIR spectroscopy are attracting attention in the fields of dermatology and cosmetology as a skin analysis tool.

The molecular structure of ECM was analyzed using FTIR spectroscopy with synchrotron radiation (SR) to obtain a high-quality spectrum with a high signal-to-noise ratio [16,17,18].

**Figure 4:**
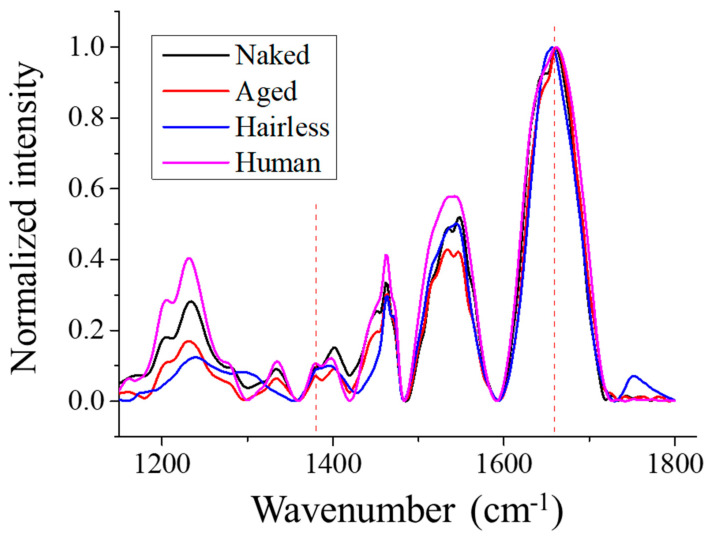
Representative FTIR spectra of the investigated skin samples. The 1386 and 1660 cm^−1^ peaks (dashed line) indicate the symmetric deformation of CH_3_ in glycosaminoglycans (GAGs) and the N–C=O stretching in protein α-helix and C=C stretching in lipids.

Figure 4 shows a comparison of typical FTIR spectra of the investigated skin samples. The strongest band located at ~1660 cm^−1^ was mainly contributed by C=O stretching in the protein α-helix, although C=C stretching in unsaturated fatty acids and other secondary protein structures may have also influenced its spectral profile [19]. A relatively weak peak was also observed at ~1386 cm^−1^ and attributed to the symmetric deformation of CH_3_ in glycosaminoglycans (GAGs), especially HA [20].

**Figure 5:**
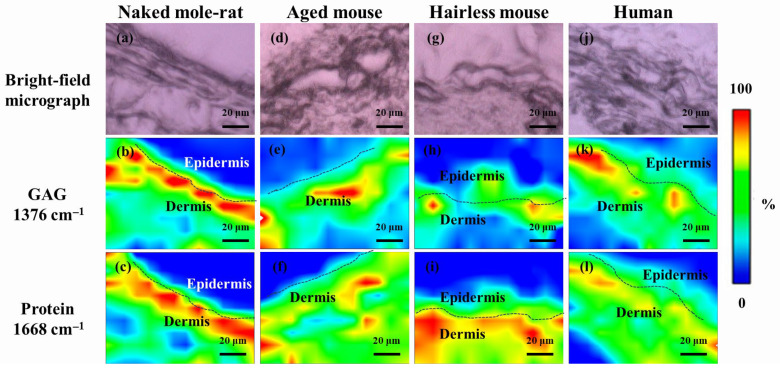
(**a**,**d**,**g**,**j**) Bright-field optical micrographs and related synchrotron radiation-based Fourier transform infrared (SR-FTIR) images of (**b**,**e**,**h**,**k**) GAGs and (**c**,**f**,**i**,**l**) protein for the investigated skin samples. The dashed lines indicate the basement membrane.

Accordingly, considering these characteristic bands, respective SR-FTIR images could be obtained using their normalized intensities. Figure 5 shows the variations in GAGs (1386 cm^−1^) and protein (1660 cm^−1^) in the depth of skin for the investigated samples. As can be seen, in the naked mole-rat, GAGs imaging revealed the presence of a tightly packed layer with high GAG and protein content which might have corresponded to the dermis. A similar phenomenon could be observed in the human skin sample, despite a relatively large intensity variation near the surface. However, for the aged mice, this layer became disconnected and discontinuous, possibly due to stratum corneum detachment. Note that, because of the nature of weak signals and the presence of noise, as well as a large probe size, intensity might have a non-zero value even at locations out of the skin.

### 2.3. Raman Analysis

**Figure 6:**
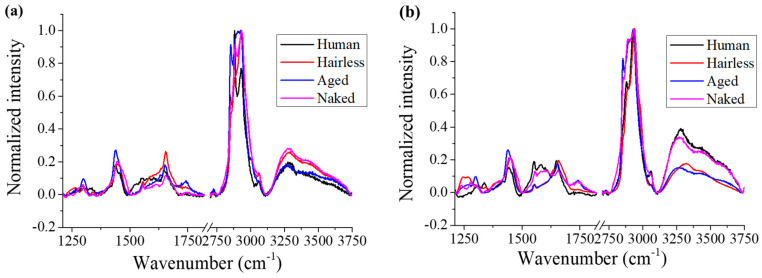
Averaged Raman spectra in the ranges (**a**) 1210–1810 and (**b**) 2720–3720 cm^−1^ for skin samples.

Figure 6 shows the averaged Raman spectra of the samples obtained in the epidermis and dermis zones, in the ranges 1210–1810 and 2720–3720 cm^−1^, respectively. As can be seen, unlike the FTIR results, the Raman spectra had significant variation in line shape, especially in the range 1210–1350 cm^−1^, in which the sharp peaks are mainly attributed to C–N stretching and C–H wagging of amide III in different protein structures; in the range 1500–1600 cm^−1^ these were mainly contributed by C=N stretching and amide II in proteins.

The GAG peak appeared at ~1380 cm^−1^ as a shoulder to the main peak at 1450 cm^−1^ induced by C–H vibrations [21]. The typical Raman spectra of HA with different molecular weights all exhibited a main peak located at approximately 1380 cm^−1^, with a similar spectral morphology despite the change in molecular weight and chain length (cf. Supplementary Materials Figure S1). The strong broad band centered at ~1660 cm^−1^ was contributed by both C=C stretching in lipids and amide I of proteins and HA and was clearly altered for skin samples because of changes in the protein secondary structure [22]. A peak at 1740 cm^−1^ could also be observed in the samples, related to C=O stretching mainly in phospholipids and cholesterol esters. C–H stretching-induced peaks in lipids and proteins are generally located in the range 2850–3100 cm^−1^ (cf. Figure 6b), while the broad band from 3200 to 3600 cm^−1^ resulted from N–H vibration and O–H stretching.

**Figure 7:**
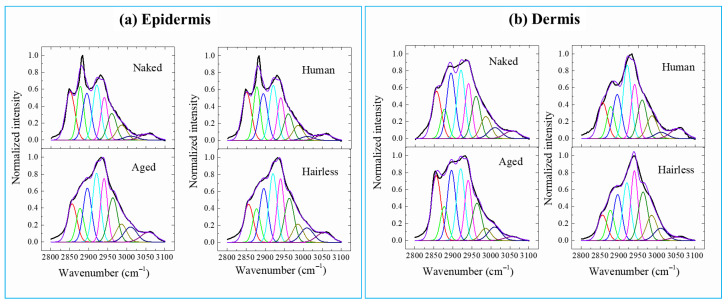
Variations in Raman spectra in the range 2720–3720 cm^−1^ obtained in the (**a**) epidermis and (**b**) dermis regions for the skin samples.

Spectral deconvolution of the amide I-related band was performed to evaluate the variations in protein secondary structure in the skin samples, as shown in Figure 7, and the assignments of relevant bands with their respective areal percentages are given in Table 1. The peaks located at ~1656, 1675 and 1690 cm^−1^ could be attributed to α-helices, β-sheets and the disordered structure of proteins [23].

As presented in Table 1, compared with the other species, the naked mole-rat exhibited a smaller fraction of α-helix, but a higher fraction of the disordered structure in the dermis zone. In the epidermis, its α-helix fraction was comparable or even higher; the disordered structure demonstrated the highest fraction. In addition, with respect to the dermis zone, the fractions of α-helix and β-sheet increased in the epidermis zone, while that of the disordered structure decreased for the naked mole-rat. The band located at ~1745 cm^−1^ was related to phospholipids, cholesterol and GAGs. Compared with the aged mice and other species, the naked mole-rats exhibited an apparently broader and asymmetric peak, as well as a peak shift in the dermis zone. In the epidermis zone, this band became rather weak, possibly because of variations in intercellular lipids and a higher contribution of HA in the dermis of naked mole-rats.

The protein structure of keratin in skin keratinocytes changes depending on the amount of water and, as water decreases, the relative proportion of α-helices increases and β-sheets decreases [24]. Older mice have a relative decrease in the proportion of β-sheets, which indicates a decrease in water content, a typical sign of aging.

**Figure 8:**
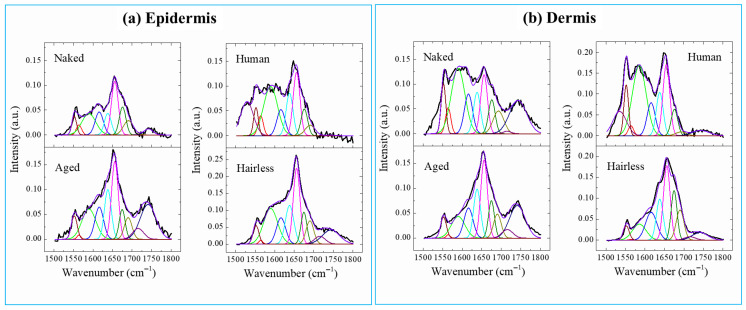
Variations in Raman spectra in the range 1500–1800 cm^−1^ obtained in the (**a**) epidermis and (**b**) dermis regions for the skin samples.

Spectral deconvolution results for the C–H broad band in the range 2800–3100 cm^−1^ in the skin samples are given in Figure 8 and Table 2. Compared with aged mice, the naked mole-rats had a lower fraction of lipid and a higher fraction of protein in both the dermis and epidermis. The fractions of the lipid bands slightly decreased in the epidermis compared with those in the dermis for the naked mole-rat but increased for aged mice.

**Figure 9:**
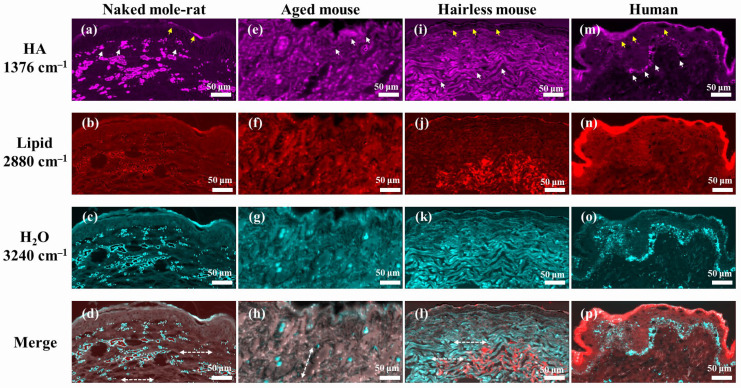
Bright-field optical micrographs with related Raman images of (**a**,**e**,**i**,**m**) HA, (**b**,**f**,**j**,**n**) lipid, (**c**,**g**,**k**,**o**) OH in water and (**d**,**h**,**l**,**p**) merged images for the investigated samples. Yellow arrows indicate stratum corneum. White arrows indicate HA just below the basement membrane. Dotted arrows indicate collagen fiber.

In the naked mole-rat, the 1389 cm^−1^ peak intensity showed a discrete stripe-like distribution parallel to the surface in the central part of the dermis, and a low value in the epidermis region, which revealed that HA had accumulated beneath the basement membrane in the dermis of naked mole-rats and was present scattered as a cluster in the central dermis, connected in an aggregated manner (cf. Figure 9a). A similar intensity variation in the 3300 cm^−1^ band could also be observed in this region, which might have been due to the presence of hydrated water. Note that HA could also be found in the stratum corneum of naked mole-rats, consistent with previous reports. Furthermore, the stratum corneum of the naked mole-rat was colocalized with water molecules and lipids, suggesting that it had a lamellar structure in which water layers and oil layers are stacked (cf. Figure 9b,c,d).

This phenomenon could not be observed in other skin samples (cf. Figure 9e–p). It was revealed that HA was universally present in the dermis of aged mice (cf. Figure 9e) while, in humans, HA was distributed beneath the basement membrane, and no HA was detected in the center of the dermis (cf. Figure 9m). Note that strong lipid bands were found in the central dermis of hairless mice (cf. Figure 9j) and in the epidermis of human skin (cf. Figure 9n).

Naked mole-rat HA forms various aggregates with a wide molecular weight distribution, and the morphology varies depending on the tissue type [8]. Naked mole-rat HA isolated from cultured skin fibroblasts and skin tissue exhibited a characteristic chain structure (chain HA), similar to the HA in the skin observed by Raman spectroscopy.

Indeed, while the collagen fibers in naked mole-rats and hairless mice were horizontally aligned, those in aged mice were randomly aligned (cf. Figure 9d,h,l). This was consistent with previous studies, which showed that collagen orientation was disrupted during aging. Furthermore, vertical collagen orientation was observed in human skin. This was probably due to differences in tissue structure depending on the location from which the skin samples were taken (human skin was taken from the abdomen, while rodent skin was taken from the dorsal region) [25].

It was difficult to determine the differences between normal tissue and pathological tissue by spectroscopic analysis from tissues composed of multiple types of molecules. The reasons for this include the enormous amount of data collected by spectroscopic analysis and the need to extract microscopic spectral changes. The overlap of the sub-bands significantly decreased the reliability for quantitative analyses without spectral deconvolution.

The Nobel Prize in Chemistry in 2024 and Physics in 2024 went to research related to artificial intelligence (AI); the introduction of AI into the field of life sciences is also expected.

The discrimination of heterogeneous sites using AI spectroscopic analysis has been reported to be effective for applications in the field of pathology, including the identification of cancer tissue [26,27,28,29]. Using AI, it may be possible to find unknown molecular groups that control hierarchical structures by extracting microscopic spectral changes that humans cannot distinguish. For spectroscopic analysis in histology, AI is expected to be used as an analytical method to systematically classify complex multivariate spectral image data [18].

Using the AI system WAVEBASE^®^, we extracted features from the entire spectrum of the skin and constructed a virtual tissue image by applying the k-means method for clustering analysis to separate the Raman spectra and visualize constituent molecules in the Raman spectra, as spatial images and respective clusters for the four types of skin samples shown in Supplementary Materials Figure S2. Accordingly, a hierarchical structure of the skin could be clearly revealed using the AI system. As Cluster 4 (orange) and Cluster 5 (yellow) emphasized the 2900 cm^−1^ protein Raman bands, and the 3300 cm^−1^ water bands, as well as the 1380 cm^−1^ HA peak, they were taken to represent collagen and hydrated water, showing a layered HA (orange) continuous distribution in the naked mole-rat, but a discrete distribution in the case of the aged mouse. Cluster 0 (red) and 1 (blue) revealed the strong band at 2860 cm^−1^, characteristic of lipid. In the naked mole-rat image, segmented by cluster of averaged Raman spectra, a unique periodic layered structure consisting of HA and lipids could be observed in the dermis, while the periodicity seemed to be longer than that of hairless mice and parallel to the surface. The skin of the naked mole-rat maintained a hierarchical structure and the ECM was composed of various molecular species (Diversity was higher in older mice compared with aged mice).

### 2.4. Optical Coherence Tomography (OCT) and Firmness Measurement

OCT, a non-destructive test similar to Raman spectroscopy, is used in the field of ophthalmology because it can observe 3D morphology.

OCT imaging is an interferometric optical scattering-based technique in which image contrast arises from refractive index differences in biological tissues governed by their dielectric response.

OCT has attracted attention in the field of dermatology and plastic surgery as a method for observing the inside of the skin without damaging it.

Yamazaki et al. used OCT to visualize the distribution of structural changes in collagen fibers in the skin [30]. There have also been attempts to construct virtual pathological images from OCT images and perform pathological diagnosis, and the range of applications of OCT is expanding yearly [31].

By combining OCT with AI, it was possible to visualize age-related changes (cell number and distribution) of stem cells inside skin [32] but, unlike Raman spectroscopy and FTIR spectroscopy, OCT could not analyze the behavior of various molecular species. This analysis technology, combining Raman and OCT, is expected to be applied to various fields such as histology and pathology in the future.

OCT microscopy can easily analyze non-invasively the topographical structure of the skin surface [33].

Morphology of the skin surface crista cutis (skin ridges) and sulcus cutis (skin furrows) has shown these to be involved in skin firmness, elasticity, and appearance [34].

**Figure 10:**
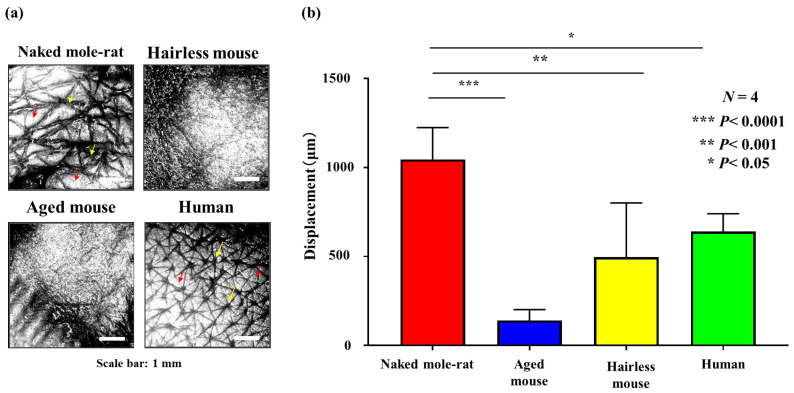
(**a**) Skin images using optical coherence tomography (OCT). Yellow arrows indicate sulcus cutis. Red arrows indicate crista cutis. (**b**) Measurement of the firmness of skin sections using compressed air (Statistical results of Tukey–Kramer multiple comparison test).

Figure 10a shows the morphology of the crista cutis and sulcus cutis by OCT imaging. The naked mole-rat crista cutis had a uniform, regular triangular pattern. Aged and hairless mice lacked a distinct skin texture, and human crista cutis were rounded and lacked a regular triangular pattern.

This en-face image, reconstructed from B-scan interferometric datasets, contained depth-resolved information derived from elastic backscattering interference and exhibited clearer visualization of wrinkle and ridge structures than conventional surface reflectance imaging due to reduced speckle noise under broadband low-coherence illumination [35,36].

Next, the firmness of the skin slices was measured using iB-Dent (Koganei; Figure 10b). When iB-Dent begins analysis, it sprays compressed air onto the object being measured and calculates the amount of indentation of the object using a laser sensor located coaxially with the compressed air nozzle. If the object being measured is hard, the amount of indentation will be small, and if the object is soft, the amount of indentation (displacement) will be large. This technique makes it possible to quantify the hardness or softness of a material. In addition, because the process measures the recovery state from the indented state over time, iB-Dent makes it possible to visually grasp transient states that had been difficult to grasp with conventional devices.

The skin firmness measurement device showed that the naked mole-rat skin sections were the most elastic of all the skin types tested. In this analysis, a high displacement indicated high elasticity.

Naked mole-rats have finely textured skin that is elastic (skin firmness), suggesting that this elasticity protects the skin from external stimuli and helps maintain a youthful and healthy appearance.

It is known that, with aging, proliferation of dermal fibroblasts and the ability to synthesize ECM components decline, resulting in a loss of flexibility and a reduction in the function of epidermal stem cells [37].

The skin of the naked mole-rats was elastic; GAGs (including HA) and collagen in the dermis played a major role in this elasticity.

HA in naked mole-rat skin formed tightly packed aggregates that have spring-like mechanical properties in addition to a strong water affinity [8]. Compared with naked mole-rat HA, HA extracted from human skin tissue did not show a supercoiled structure and was significantly more rigid.

Furthermore, we reported that the highly folded structure of naked mole-rat HA contributed to both its elasticity and youthfulness. These reports strongly supported the validity of our hypothesis.

### 2.5. Discussion

Symptoms associated with skin aging, such as abnormal skin moisture content, collagen or elastin degeneration, uneven smooth-texture, and decreased elasticity, have, in recent years, been referred to as “skin frailty” [38].

Preventing skin frailty can help prevent skin tears and lead to a longer, healthier life. Wild naked mole-rats live underground, where their skin is rarely exposed to ultraviolet light. Also, as they move through narrow tunnels, their skin constantly rubs against the tunnel walls. It is thought that the high elasticity of naked mole-rat skin prevents damage from rubbing against the tunnel walls [39]. Although research into hyaluronic acid as an anti-aging and anti-tumor factor in naked mole-rats has been reported [3,4], there have been few studies that have investigated the localization of HA in skin tissue or the surface structure of the skin of naked mole-rats.

The dermis is composed of collagen, elastin, HA, and proteoglycans, which maintain the elasticity of the skin. Approximately 70% of the dermis is collagen. It has been reported that type I collagen degradation accelerates and synthesis decreases with aging, and this decrease in collagen leads to dermal atrophy [40,41].

HA and proteoglycans are water-retaining components that store large amounts of water and moisturize the skin.

In youthful skin with “skin firmness,” these components are produced in sufficient quantities so that the structure that supports the skin’s elasticity is maintained.

HA is composed of alternating bonds between *N*-acetylglucosamine and glucuronic acid that become entangled and bond with each other in water to form a viscous aqueous solution. HA is an extremely large molecule among biopolymers and its synthesis decreases with age and decreases rapidly in older people.

GAGs have a high binding capacity with water, and there is a high correlation between the content and distribution of GAGs and water in the dermis. Therefore, the Raman results also showed that water and HA colocalized [42].

Age-related hardening of the dermis is induced by a decrease in the number of blood vessels; hardening of the dermis leads to a decrease in the function of epidermal stem cells [37]. It has been speculated that hardening of the dermis in aged mice is due to a decline in stem cell function and reduced turnover.

Miura et al. reported that the addition of sulfated HA maintains the undifferentiated state of human iPS cells, making it useful for regenerative medicine and cell therapy [43]. The HA clusters just below the basement membrane of naked mole-rats are thought to be involved in dermis flexibility and in the differentiation and stemness of epidermal stem cells.

HA has different molecular weight forms depending on the layer of skin in which it is present. In the epidermis layer, high concentrations of high-molecular-weight HA retain moisture and ions in the gaps between cells; low-molecular-weight HA enhances moisture retention in the stratum corneum and high-molecular-weight HA is broken down into intermediate-sized HA in the dermis layer.

Hyaluronic acid present in the stratum corneum of naked mole-rats is thought to play an important role in preventing dryness and in moisturizing the skin.

Tensile stress in the skin is believed to dehydrate fluid inside the skin, reducing its weight and volume and inducing changes in skin structure (flattening of the boundary between the epidermis and dermis and a decrease in collagen) [44]. It is thought that the HA clusters in naked mole-rats maintain moisture and prevent changes in the skin structure. The boundary between the epidermis and dermis of naked mole-rats was uneven, the skin was elastic, and no obvious structural changes were observed.

Epidermal metabolism is essential for maintaining skin barrier function; it is important to control the proliferation and differentiation of epidermal stem cells located in the basal layer of the epidermis.

When a single epidermal stem cell located on the basement membrane differentiates, neighboring cells proliferate and replenish, maintaining homeostasis [45].

Raja et al. reported that the properties of epidermal stem cells are impaired with age, and that rapidly the dividing epidermal stem cells gradually decrease [6].

It is believed that the dermis that backs the epidermis hardens with age, inducing epidermal stem cell aging and causing a decline in function. In a simulation of aging of skin structure by Ohno et al. [7], when the dermis was hardened, deformation of the basement membrane became flatter and the epidermis became thinner, which was consistent with the histological findings of aged mice (flattening of the basement membrane and thinning of the epidermis).

Epidermal stem cells in the basal layer of the skin have a strongly activated endoplasmic reticulum stress response pathway, which is believed to maintain “youth” (proliferative activity). The ultra-high-molecular-weight hyaluronic acid produced by naked mole-rats is believed to mask the HA receptor CD44 and inhibit its downstream p53 signaling by interfering with protein–protein interactions, thereby exerting a cytoprotective effect [46].

It is possible that the HA in the dermis of naked mole-rats provides elasticity, promotes the turnover of epidermal stem cells, and maintains barrier function.

It has been speculated that the maintenance of stem cell function in naked mole-rat skin is influenced by multiple factors, such as the endoplasmic reticulum stress response [47], clearance of senescent cells [48] and mechanosensory channels [37].

The enzymatic activity of hyaluronic acid-degrading enzymes in naked mole-rats is reduced [49,50], and it is believed that high-molecular-weight HA accumulates in the body.

It has been speculated that high-molecular-weight HA in the naked mole-rat dermis is maintained without being broken down. In mice, HA is broken down by hyaluronan-degrading enzymes and intermediate-molecular-weight HA is distributed [51]. Raman spectroscopy showed that HA is ubiquitous in the mouse dermis whereas, in naked mole-rats, HA is distributed in clusters, forming a network that fills the tissue gaps. The biological activity of hyaluronan-degrading enzymes must be interpreted with caution as mouse models do not resemble those in humans.

### 2.6. Limitations

The naked mole-rats used in this study are rare animals, and the number of samples was limited. Skin firmness measurements and OCT analysis were also performed using skin slices. It is known that a decrease in blood vessels due to aging affects dermal hardness, but this study was unable to take the influence of blood vessels into account. Future work will require conducting and verifying these non-destructive tests on living skin.

Generally, the skin texture grooves are believed to coincide with the protrusions of the basement membrane.

Because hairless and aged mice lack skin texture structure, careful consideration must be given to the animal species when evaluating skin texture structure and histological morphology.

Furthermore, while the shape of the skin texture in naked mole-rats and humans was relatively similar, the skin ridges in naked mole-rats were larger than in humans. Generally, a finer texture is considered to indicate a softer, smoother, more elastic, and more youthful skin. However, there was no complete correlation between surface skin texture (in millimeters), histological findings (in micrometers), and physical properties (hardness), revealing a gap.

Ohashi et al. reported using axolotls in which keratinocytes are the main source of collagen in the dermis [52]. Fibroblasts present in the dermis produce additional collagen, reinforcing and repairing the collagen fibers produced by keratinocytes. This collagen production mechanism is likely to have been conserved in other animal species such as zebrafish, chicken, and mouse.

Naked mole-rats had relatively higher HA and protein (collagen) contents compared with aged mice but, unlike pellet-fed mice, captive naked mole-rats also ate vegetables and fruits, which may have affected collagen and HA contents.

The three-dimensional meshwork structure of skin collagen is thought to be important for skin texture and firmness, and future research into the skin structure of naked mole-rats at the nano-level is needed.

The naked mole-rats used in this study were classified as young, at 54 weeks of age, so when comparing them with other rodents, it was important to organize them based on the relative aging stages rather than comparing absolute ages.

Naked mole-rats are extremely long-lived rodents that do not appear to age [53], but aging at the molecular level certainly occurs and can be measured by epigenetic changes [54].

By measuring the ages estimated by methylation levels (DNA methylation clocks) of naked mole-rats, mice, and humans, age matching them by epigenetic age, and taking into account the degree of aging (biological aging), accurate comparisons of aging across species were possible.

Although mice and naked mole-rats are of similar size, they are phylogenetically very distant, so care must be taken when interpreting the data.

Because naked mole-rats are rare and it was difficult to secure a sufficient sample size, future research will focus on reproducing these pathological conditions through simulations using mathematical skin models to fill this measurement gap [7].

## 3. Conclusions

Chen et al. reported that amino acid substitutions in naked mole-rat cyclic GMP-AMP synthase (cGAS) prolong chromatin binding after DNA damage and promote homologous recombination-mediated DNA repair [55]. Such properties of cGAS may contribute to enhanced genome maintenance and stress resistance in naked mole-rats. However, genetic analysis of naked mole-rat resistance to aging has its limitations [56], and therefore comprehensive analysis of the physical properties and morphology of the ECM is necessary.

The following differences were confirmed between the skin of naked mole-rats and aged mice:

1. The epidermis of naked mole-rats was thickened, and the stratum corneum was maintained. In contrast, the epidermis of aged mice was thinned, and the stratum corneum had fallen off.
2. The texture and elasticity of naked mole-rat skin were maintained, while the skin of aged mice was hardened. This suggested that HA clusters just beneath the dermal basement membrane of naked mole-rats contributed to elasticity and anti-aging.

Combining Raman spectroscopy with AI made it possible to rapidly obtain virtual pathology images, and there have been attempts to apply this to pathology diagnosis. Raman spectroscopy and OCT, which enable non-staining and non-invasive “molecular” and “morphological” analysis, are promising pathological diagnostic tools and are thought to complement each other’s shortcomings.

The two core technologies of this research, “anti-aging model: naked mole-rats” and “optical analysis,” can be applied to clarifying and diagnosing the pathology of various skin diseases, and are expected to have a ripple effect not only in dermatology but also in various other fields such as cosmetics and therapeutic gel scaffolds.

## 4. Materials and Methods

### 4.1. Animals

The Ethics Committees of Kyoto Prefectural University of Medicine and Kumamoto University approved all procedures, in accordance with the Guide for the Care and Use of Laboratory Animals (United States National Institutes of Health, Bethesda, MD, USA). The animal study protocol was approved by the Institutional Review Board of Kyoto Prefectural University of Medicine (codes M2025-297, M2025-521; 1 April 2025, 1 July 2025) and Kumamoto University (codes A2020-042, A2022-079, and A2024-063; 1 April 2020, 20 July 2022).

The naked mole-rats and aged mice used in this study were 54-week-old females. Naked mole-rats were maintained in Kumamoto University (Kumamoto, Japan). C57BL/6 mice were purchased from CLEA Japan, Inc. (Meguro, Tokyo, Japan). Rodent (naked mole-rats and aged mice) skin was taken from the dorsal region.

Hairless mice show a phenotype of hair loss at about 2 weeks of age. Hairless mouse skin (7 weeks old, female) taken from the dorsal region was purchased from Hoshino Laboratory Animals, Inc. (Bando, Ibaraki, Japan).

Skh:HR mice (genetics: Hr^hr^/Hr^hr^) were generated by crossing hairless mice with CBA mice [57]. Albino mice were selected from Skh:HR mice and bred Specific Pathogen Free (SPF) (Hos:HR-1). Skin from the backs of Hos:HR-1 mice was purchased and used as a control.

Human skin (White, 28-year-old and 35-year-old women) taken from the abdominal region was purchased from KAC Co., Ltd. (Nakagyo, Kyoto, Japan).

### 4.2. Histochemical Analyses

Diameter 8–10 mm rims of skin tissue were taken. Formalin-fixed skin samples were embedded in paraffin, sliced at 4 μm thickness using a microtome. To perform H&E staining, sections (10 μm) were deparaffinized, stained with Cole’s Hematoxylin Solution (Muto Pure Chemicals Co. Ltd., Bunkyo, Tokyo) for 15 min and washed with deionized water. After washing, the sections were immersed in 1% hydrochloric acid–ethanol solution, then stained with eosin and subsequently rinsed in 99.5% ethanol.

Because the G1 domain of Versican (Proteoglycan) selectively binds to HA but not to other GAGs, it serves as a useful tool for detecting HA16]. Sections were stained using a biotinylated HA-binding protein (Biotin-HABP) probe. Skin tissue sections were then treated with 0.3% hydrogen peroxide in methanol and washed with phosphate-buffered saline (PBS) (Nacalai Tesque, Inc., Nakagyo, Kyoto, Japan) three times. After washing, sections were blocked in Avidin/Biotin Blocking solution (Nichirei Biosciences Inc., Chuo, Tokyo, Japan) for 15 min at room temperature. Biotin-HABP <Catalog No.: BC41> (Cosmo Bio Company, Limited, Koto, Tokyo, Japan, 1:833 dilution) in PBS was then added, and the slides were placed in a humidity chamber for 15 min at room temperature and washed with PBS three times. After washing, the tissue sections were then incubated for 10 min with peroxidase-conjugated streptavidin (Nichirei Biosciences Inc.) and washed with PBS three times. The sites of peroxidase activity were determined using diaminobenzidine (DAB; Dako EnVision™ detection system, Agilent Dako, Santa Clara, CA, USA) as the substrate for 10 min and washed with deionized water. Counter staining was performed for nucleus with Mayer’s hematoxylin solution (Muto Pure Chemicals Co., Ltd.).

As a positive control, rat skin (tissue that can be clearly stained with Alcian blue (pH 2.5)) was used in advance. HA is known to be present in various organs and tissues, so hyaluronidase-digested tissue was used as a negative control, based on the conditions obtained in a separate hyaluronidase digestion test. Denatured collagen was detected using a Collagen Hybridizing Peptide kit (Collagen Hybridizing Peptide Biotin Conjugate, 3Helix Inc., Salt Lake City, UT, USA), according to the manufacturer’s instructions [58].

After the staining procedure, the sections were observed under a fluorescence microscope (BZ-X710, Keyence, Osaka, Japan).

### 4.3. Synchrotron Radiation-Based Fourier Transform Infrared Spectroscopy

Tissue preparation was also carried out for the analysis of Fourier transform infrared (FTIR) spectroscopy. To investigate the structure and distribution of collagen and GAGs in the ECM, Synchrotron Radiation-based (SR)-FTIR was conducted using the Beamline43 (43IR) at a synchrotron radiation facility, SPring-8 (Sayo, Hyogo, Japan), as the procedures for the preparation of skin samples and the spectral analysis are given in the following.

Briefly, the skin tissues were embedded in a compound with an optimal cutting temperature, then flash-frozen, and finally sliced sagittally at 5 μm thickness from the frozen blocks using a cryostat. Frozen sections (5 µm thick) were thaw-mounted onto the substrates of glass slides or barium fluoride (BaF_2_) (Pier Optics Co., Ltd., Gunma, Japan).

The FTIR spectra were collected using the Hyperion 2000 (Bruker Corporation, Billerica, MA, USA). The experimental conditions for the analyses included: (i) a 250 × 250 μm^2^ liquid MCT/A detector cooled by nitrogen, (ii) a Schwarzschild objective lens with a magnification of 32× and a numerical aperture of 0.65, (iii) a knife-edge aperture controlled by computer, (iv) a 3-dimensional movable stage combined with a VERTEX 70 spectrometer (Bruker Corporation) and (v) a Michelson interferometer. The spectral resolution was 0.4 cm^−1^ and the spatial resolution was around 10 μm. The main skin components, such as GAGs, α-helices and random coils, were calculated and compared between both groups. For GAGs and proteins, integrated areas were assessed at spectral positions 1376 and 1660 cm^−1^, respectively [16,18].

### 4.4. Raman Spectroscopy

Spectroscopic imaging of the investigated skin tissues was taken by a Raman microscope (RAMANtouch, Bruker Company/Nanophoton, Osaka, Japan). The investigated area on the samples was selected to be the same as the stained images shown above, and for each sample around 400 × 200 images were collected, with a laser exposure time of 30 s for each line (400 points). A commercially available software (Raman viewer 1.4., Nanophoton, Osaka, Japan) was used for the spectral analysis, and the method of singular value decomposition (SVD) was used for denoising the spectra. In addition, contrast imaging was applied for visualizing the compositional distribution, taking advantage of the marker bands, such as the 1123 cm^−1^ band that indicated HA, the 2880 cm^−1^ band that indicated lipid, and the 3200 cm^−1^ band that indicated H_2_O.

### 4.5. Chemometric Analysis

We attempted chemometric analysis using unsupervised learning based on AI as a means of efficiently extracting information from spectroscopic images [18]. Raman hyperspectral imaging data were analyzed by projecting the spectra into a latent space using uniform manifold approximation and projection (UMAP), followed by k-means cluster analysis. With this data processing, spatially resolved segmented spectroscopic images of skin tissue are obtained. From the averaged FTIR spectra of each specimen, the second derivative was calculated and used as input data for the UMAP multivariate method using the Material Informatics software WAVEBASE 1.0 (Toyota Motor Corporation, Toyota, Aichi, Japan). WAVEBASE contains function for hyperspectral data processing with unsupervised learning by UMAP and clustering automatically. One can set an arbitrary number for clusters for segmenting an image. In this case, we applied class as seven, and calculated an averaged spectrum for each cluster.

### 4.6. Skin Firmness Measurement

Skin firmness was measured using an iB-Dent (Koganei Corporation, Koganei City, Tokyo). The skin section was placed in the iB-Dent and compressed air (pressure 200 kPa) (Supplementary Materials Figure S3) was blown from above to measure the displacement. The displacement was quantified and calculated using a dedicated application. Skin firmness analyses are presented as a mean value ± standard deviation of three independent experiments (*n* = 4).

### 4.7. Optical Coherence Tomography (OCT)

OCT imaging was performed using a microscope-type low-coherence interferometric OCT system (Tatsuta Electric Wire & Cable Co., Ltd., Kyoto, Japan) equipped with a broadband superluminescent diode (SLD) light source centered at approximately 840 nm with a broadband spectral bandwidth of approximately 100 nm.

The sample arm was configured in a microscope geometry and focused onto the skin surface using a long-working-distance 10× Mitutoyo Plan Apochromat near-infrared (NIR) objective lens (MY10X-823, Mitutoyo Corp., Kawasaki, Japan) with a numerical aperture of 0.26 and a working distance of 31 mm, designed for broadband visible to near-infrared operation (480–1800 nm).

### 4.8. Statistical Analysis

Data were tested for significance using the Tukey–Kramer multiple comparison test and Prism software GraphPad Software 10 (GraphPad Software, Inc., Boston, MA, USA). *p*-values < 0.01 were considered statistically significant and are labeled with two asterisks.

## Figures and Tables

**Table 1:** Areal percentages of deconvoluted Raman bands of amide I and their assignments for the investigated skin samples.

Peak (cm^−1^)	Assignment	Percent in Dermis (%)				Percent in Epidermis (%)			
		Human	Hairless	Aged	Naked	Human	Hairless	Aged	Naked
1638	Amide I, *v*(C=O) in protein segment	28.3	21.5	26.6	24.6	29.0	27.1	28.3	17.6
1656	Amide I, *v*(C=O) in α-helix	43.5	38.5	41.6	34.9	43.1	42.5	43.4	43.6
1675	Amide I, *v*(C=O) in β sheet	17.6	22.4	17.4	19.3	17.4	15.6	14.5	21.8
1690	Amide I, *v*(C=O) in disordered structure	10.7	17.7	14.4	21.2	10.5	14.8	13.8	17.0

**Table 2:** Areal percentages of deconvoluted C–H stretching-induced Raman bands in protein and lipid and their assignments for the investigated skin samples.

Peak (cm^−1^)	Assignment	Percent in Dermis (%)				Percent in Epidermis (%)			
		Human	Hairless	Aged	Naked	Human	Hairless	Aged	Naked
2856	*vs*(CH_2_) in lipid (liquid)	12.5	8.9	19.5	14.9	17.8	12	20.8	13.9
2877	*vs*(CH_3_) in lipid	8.9	8.4	8.1	7.3	14.3	8.5	9.0	7
2894	*v*(CH) in protein & lipid	13.8	14.2	18.7	18.6	15.6	15.1	19.1	17.6
2920	*v*_as_(CH_2_) in cholesterol & phospholipids and *v*(CH) in HA	21.6	17	18.3	18.2	17.2	18.4	18.4	17.7
2940	*v*_as_(CH_2_) in protein	13.9	17.8	13.2	12.7	11.6	14.7	11.6	12.6
2960	*v*_as_(CH_3_) in protein and lipid (out-of-plane chain end)	12.6	16.8	11.1	12.5	11.9	13.9	9.6	12.6
2986	CH_α,α_, stretching	9.2	9.8	4.2	8	6.1	6.3	2.7	8.1
3010	*v*(C=C-H) in lipid chains	1.6	5.5	5.1	3.7	2	6.2	5.7	5.1
3050	*v*(=C-H) aromatic stretching in lipids	5.9	1.7	1.7	4.1	3.7	5	3.1	5.4

## Data Availability

The original contributions presented in this study are included in the article/Supplementary Materials; further inquiries can be directed to the corresponding author.

## Supplementary Materials

The following supporting information can be downloaded at: https://www.mdpi.com/article/10.3390/gels12040303/s1, Figure S1: The average spectrum of HA standard samples was obtained; Figure S2: Averaged spectra of the clusters were obtained using unsupervised learning based on AI; Figure S3: Depiction of skin firmness measurement for the study.

## Abbreviations

The following abbreviations are used in this manuscript:

AI	artificial intelligence
CHP	collagen hybridizing peptide
FFA	free fatty acids
H&E	hematoxylin and eosin
HA	hyaluronic acid
ISIR	Institute of Scientific and Industrial Research
NMF	natural moisturizing factor
OCT	optical coherence tomography
SPF	Specific Pathogen Free
SR	synchrotron radiation
SVD	singular value decomposition
UMAP	uniform manifold approximation and projection
VOCT	vibrational optical coherence tomography
