# Supplementary material for: Spectroscopic Analysis of the Extracellular Matrix Hierarchical Structure in Naked Mole-Rat Skin
Source: Gels. 2026 Apr 1;12(4):303. doi: 10.3390/gels12040303 (PMC13116378; doi:10.3390/gels12040303)
Supplement: Supplementary file 1 [file gels-12-00303-s001.zip › gels-4182358-supplementary.pdf]

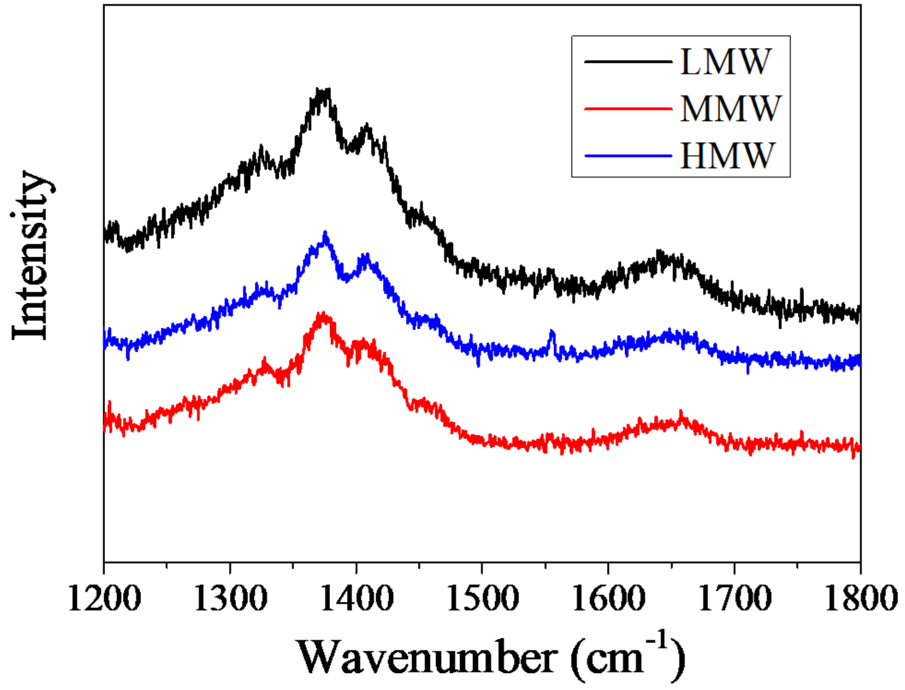

Supplementary Materials Figure S1.

Typical Raman spectra of high-molecular-weight (HMW, 1.6–2.5 million) HA, median-molecular-weight (MMW, 100–300 thousand) HA, and low-molecular-weight (LMW, 2827) HA in the range 1200–1800 cm^{-1}. All HA standards were purchased from PG Research (Kodaira City, Tokyo, Japan).

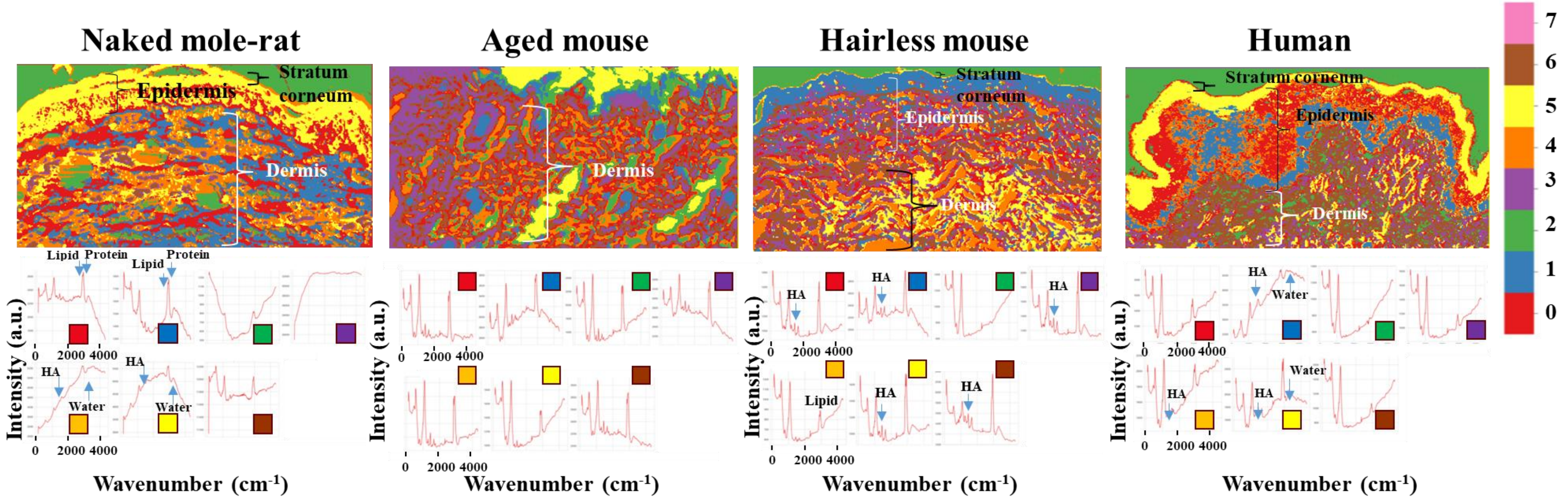

Supplementary Materials Figure S2.

A hyperspectral image constructed from Raman spectra was subjected to dimensionality reduction using UMAP, after which the UMAP-embedded space was k-means clustered to generate a segmented image. In the Naked mole-rat image, segmented by clustering averaged Raman spectra, one could observe in the dermis a unique periodic laminar structure consisting of HA and lipid. Averaged spectra of the red and blue areas exhibited strong signals, a 2860 cm^{-1} peak, attributed to the lipid, orange and yellow areas containing HA, a 1380cm^{-1} peak, and water, 3240cm^{-1} and broad fluorescence. Periodicity seemed to be longer than for Hairless mice and parallel to the surface. Spectrum imaging emphasized the structural characteristics of the samples.

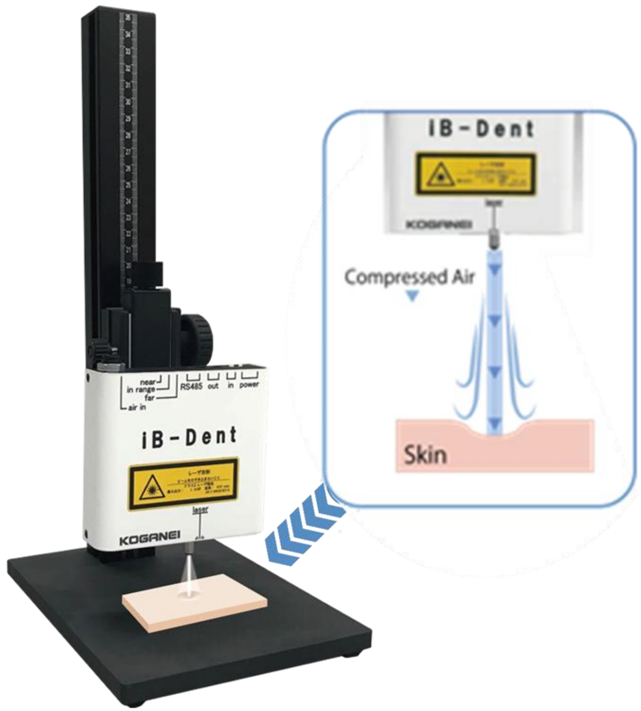

iB-Dent (Koganei)

Supplementary Materials Figure S3.

Depiction of skin firmness measurement for the study.
